# Assessment of Management to Mitigate Anthropogenic Effects on Large Whales

**DOI:** 10.1111/j.1523-1739.2012.01934.x

**Published:** 2012-10-01

**Authors:** Julie M Van Der Hoop, Michael J Moore, Susan G Barco, Timothy VN Cole, Pierre-Yves Daoust, Allison G Henry, Donald F McAlpine, William A McLellan, Tonya Wimmer, Andrew R Solow

**Affiliations:** * Biology Department, Woods Hole Oceanographic InstitutionWoods Hole, MA 02543, U.S.A.; ‡ Virginia Aquarium & Marine Science Center, Research & Conservation DivisionVirginia Beach, VA 23451, U.S.A.; § NOAA National Marine Fisheries Service, NEFSCWoods Hole, MA 02543, U.S.A.; ** Department of Pathology & Microbiology, Canadian Cooperative Wildlife Health Centre, Atlantic Veterinary College, University of Prince Edward IslandCharlottetown, Prince Edward Island C1A 4P3, Canada; †† New Brunswick MuseumSaint John, New Brunswick E2K 1E5, Canada; ‡‡ Biology and Marine Biology, University of North Carolina WilmingtonWilmington, NC 28403, U.S.A.; §§ Marine Animal Response Society, Nova Scotia MuseumHalifax, Nova Scotia B3H 4J1, Canada; *** Marine Policy Center, Woods Hole Oceanographic InstitutionWoods Hole, MA 02543, U.S.A.

**Keywords:** entanglement, evaluation of management/mitigation efforts, human-interaction, large whales, mortality, necropsy, vessel-strike, Ballenas mayores, enmarañamiento, evaluación de esfuerzos de manejo/mitigación, impacto con embarcaciones, interacción humana, mortalidad, necropsia

## Abstract

**Abstract:**

United States and Canadian governments have responded to legal requirements to reduce human-induced whale mortality via vessel strikes and entanglement in fishing gear by implementing a suite of regulatory actions. We analyzed the spatial and temporal patterns of mortality of large whales in the Northwest Atlantic (23.5°N to 48.0°N), 1970 through 2009, in the context of management changes. We used a multinomial logistic model fitted by maximum likelihood to detect trends in cause-specific mortalities with time. We compared the number of human-caused mortalities with U.S. federally established levels of potential biological removal (i.e., species-specific sustainable human-caused mortality). From 1970 through 2009, 1762 mortalities (all known) and serious injuries (likely fatal) involved 8 species of large whales. We determined cause of death for 43% of all mortalities; of those, 67% (502) resulted from human interactions. Entanglement in fishing gear was the primary cause of death across all species (n = 323), followed by natural causes (n = 248) and vessel strikes (n = 171). Established sustainable levels of mortality were consistently exceeded in 2 species by up to 650%. Probabilities of entanglement and vessel-strike mortality increased significantly from 1990 through 2009. There was no significant change in the local intensity of all or vessel-strike mortalities before and after 2003, the year after which numerous mitigation efforts were enacted. So far, regulatory efforts have not reduced the lethal effects of human activities to large whales on a population-range basis, although we do not exclude the possibility of success of targeted measures for specific local habitats that were not within the resolution of our analyses. It is unclear how shortfalls in management design or compliance relate to our findings. Analyses such as the one we conducted are crucial in critically evaluating wildlife-management decisions. The results of these analyses can provide managers with direction for modifying regulated measures and can be applied globally to mortality-driven conservation issues.

Evaluación del Manejo para Mitigar Efectos Antropogénicos sobre Ballenas Mayores

**Resumen:**

Los gobiernos de Estados Unidos y Canadá han respondido a requerimientos legales para reducir la mortalidad de ballenas inducida por humanos por medio de impacto con embarcaciones y enmarañamiento en artes de pesca mediante la implementación de un conjunto de acciones reguladoras. Analizamos los patrones espaciales y temporales de la mortalidad de ballenas mayores en el Atlántico Noroccidental (23.5°N a 48.0°N), de 1970 a 2009, en el contexto de cambios de manejo. Utilizamos un modelo logístico multinomial ajustado por la máxima probabilidad de detección de tendencias en mortalidades por causa específica en el tiempo. Comparamos el número de muertes provocadas por humanos con los niveles de remoción biológica potencial (i.e., mortalidad específica provocada por humanos sustentable). De 1970 a 2009, hubo 1762 muertes (conocidas) y lesiones serias (casi fatales) involucrando 8 especies de ballenas mayores. Determinamos la causa de 43% de todas las muertes; de ellas, 67% (502) resultaron de interacciones humanas. El enmarañamiento en artes de pesca fue la causa principal de muerte en todas las especies (n = 323), seguida de causas naturales (n = 248) e impacto de embarcaciones (n = 171). Los niveles sustentables de mortalidad establecidos fueron excedidos consistentemente hasta en 650% en 2 especies. Las probabilidades de muerte por enmarañamiento y por impacto de embarcaciones incrementaron significativamente de 1990 a 2009. No hubo cambio significativo en la intensidad local de mortalidad por todas las causas o por impacto de embarcaciones antes y después de 2003, año en el que se implementaron numerosos esfuerzos de mitigación. Hasta ahora, los esfuerzos regulatorios no han reducido los efectos letales de las actividades humanas sobre las ballenas a nivel de población, aunque no excluimos la posibilidad de éxito de medidas enfocadas a hábitats locales específicos que no estuvieron dentro de la resolución de nuestro análisis. No es claro como se relacionan con nuestros resultados las deficiencias en el diseño o implementación del manejo. Análisis como el que realizamos son cruciales para la evaluación crítica de decisiones para el manejo de vida silvestre, y los resultados de estos análisis pueden proporcionar directrices a los manejadores para que modifiquen medidas regulatorias y puedan ser aplicadas globalmente en temas de conservación relacionadas con mortalidad.

## Introduction

Six of 8 populations of large whales in the Northwest Atlantic are endangered or of special concern under existing U.S. and Canadian legislation (Table 1). Yet, many of these managed populations have not recovered (Clapham et al. 2009; Fujiwara & Caswell 2011) and have high mortality rates, especially from vessel strikes and entanglement in fishing gear (Volgenau et al. 2012; Laist et al. 1985).

**Table 1:** Population status, listing date, and current potential biological removal (PBR) (calculated for transborder species range estimates) for large whale species inhabiting coastal eastern North America (PBR values from Waring et al. 1995)

*Common and scientific name*	*Population status in Canada (authority, listing year)*^a^	*Population status in United States (authority, listing year)*^b^	*Minimum population estimate (stock, year)*	*Current PBR (stock, year)*
Blue whale *Balaenoptera musculus*	Endangered (SARA, 2005)	Endangered (ESA, 1970)	440 (2009)	0.9 (2010)
Bryde's whale *Balaenoptera edeni*	Not assessed	Not listed (data deficient)	5 (Gulf of Mexico stock, 2007)	0.1 (2009)
Fin whale *Balaenoptera physalus*	Special concern (SARA, 2006)	Endangered (ESA, 1970)	3269 (2007)	6.5 (2010)
Humpback whale *Megaptera novaeangliae*	Not at risk^d^ (COSEWIC, 2003)	Endangered (ESA, 1970)	549^c^ (2006)	1.1 (2010)
Minke whale *Balaenoptera acutorostrata*	Not at risk^d^ (COSEWIC, 2006)	Not listed	6909 (2007)	69 (2010)
North Atlantic right whale *Eubalaena glacialis*	Endangered (SARA, 2005)	Endangered (ESA, 1970)	461 (2009)	0.7 (2010)
Sei whale *Balaenoptera borealis*	Data deficient^d^ (COSEWIC, 2003)	Endangered (ESA, 1970)	208 (2004)	0.4 (2010)
Sperm whale *Physeter macrocephalus*	Not at risk^d^ (COSEWIC, 1996)	Endangered (ESA, 1970)	3539 (Atlantic, 2004)	7.1 (Atlantic, 2007)
			1409 (Gulf of Mexico, 2003–2004)	2.8 (Gulf of Mexico, 2010)

a Species at Risk Act (SARA) and Committee on the Status of Endangered Wildlife in Canada (COSEWIC).

b United States Endangered Species Act (ESA).

c As is assumed for analyses conducted by federal agencies (e.g., Glass et al. 2010), we assumed all humpbacks occurring in or near U.S. and southeast Canadian waters involved the Gulf of Maine stock.

d Populations assessed by COSEWIC as not at risk or data deficient are not required to be considered for listing under SARA.

All species of large whales appear to be susceptible to vessel strikes, although high encounter probability suggests mortality may be more prevalent in species occupying areas with higher levels of vessel traffic (van der Hoop et al. 2010). Fin whales (*Balaenoptera physalus*) are the most commonly reported species (29.2%) in the current global vessel-strike data set maintained by the International Whaling Commission, followed by humpback (*Megaptera novaeangliae*) (21.2%) and North Atlantic right whales (*Eubalaena glacialis*) (hereafter right whales) (16.0%) (Van Waerebeek & Leaper 2008). However, when placed in context of population size, right whales are 2 orders of magnitude more prone to vessel strikes (Vanderlaan & Taggart 2004).

Results of analyses of scar occurrence on whales indicate 82% of right whales and 48-57% of humpback whales in the Gulf of Maine have had at least one previous entanglement in fishing gear (Robbins & Mattila 2012; Knowlton et al. 2000). Entanglement of large whales may result in death of individuals anchored in gear or individuals breaking free either cleanly or carrying all or a portion of the entangling lines (Clapham et al. 2009). Trailing gear can create severe drag and may inhibit foraging ability and thus lead to starvation. A whale can remain entangled for months to years (Moore et al. 2006; NARWC 2011).

The governments of Canada and the United States are required by law to reduce human-induced mortality by vessel strikes and entanglement in fishing gear to assist species recovery (NOAA 1997; NMFS 2004; Brown et al. 2009). Numerous mitigation efforts have been implemented at state and federal levels (Supporting Information), and some federal efforts have been adopted by international agencies (e.g., International Maritime Organization [IMO]).

Management efforts to mitigate anthropogenic effects on endangered species require ongoing assessment. Current tools to assess the efficacy of such actions are limited (Pace 2011), and the effects of management should be reflected in reduced numbers and causes of mortalities over time. Although many management plans have been developed and implemented specifically for right whales, it is often, perhaps incorrectly, assumed that other large whale species will also benefit. We included 8 species of large whales in our analyses because sample size limits determination of success of a given management action to right whales alone. Although these species all inhabit the study area for all or a portion of their lives, they exhibit ecological differences. Management actions aimed at one species may not provide similar benefits to other species. Similarly, a substantial reduction in mortality of other, nontarget large whales does not mean mortality of the target species is reduced.

Data compiled from observed strandings and sightings of dead or seriously injured whales in Canada and the United States provide a sample with which to study whale mortality. Data collected in over 60 countries (IWC 2011) is available and can be used to evaluate changes in relative spatial and temporal patterns of whale mortality following major regulatory and management changes; assess the sustainability of takes of specific endangered species compared with federally established values; and identify species, regions, or age classes specifically at risk.

We summarized all known mortalities and serious injuries (defined below) to 8 species of large whales along the U.S. East Coast and Canadian Maritimes from 1970 through 2009 and examined their spatial, temporal, and cause-specific trends to determine whether mitigation has been successful in reducing human-induced mortality to these species and to inform future regulations.

## Methods

### Data Acquisition

We compiled reports of large-whale strandings, mortalities, and necropsies that occurred between 23.5°N and 48.0°N latitude from the coast to continental shelf, from 1970 through 2009. We obtained Canadian records from the Marine Animal Response Network of Atlantic Canada (Nemiroff et al. 1994) and American records from the National Oceanic and Atmospheric Administration (NOAA) Southeast and Northeast US Marine Mammal Stranding Network Databases, to which all strandings and sightings are reported through local response programs; NOAA Northeast Science Fisheries Center; NOAA National Marine Mammal Health and Stranding Response National Database; North Atlantic Right Whale Consortium Database; and Smithsonian National Museum of Natural History Division of Mammals Collections Database. We completed data management and archiving in Microsoft Access.

From these records, we compiled field identification number(s), taxon, date, location, sex, length, and presumed cause of death. We left fields blank if information was missing. Species identification was provided in initial reports.

We standardized total length measurements to centimeters and latitude and longitude coordinates (where an animal was first reported dead) to decimal degrees. If absent (104 cases, 5.9%), we estimated coordinates from locations provided (e.g., 3 miles south of Ocracoke Inlet, North Carolina).

We assigned age class by species-specific age-length relations (Kraus 2011; Geraci & Lounsbury 1989) unless age was known from catalogue identification or maturity confirmed by evidence of pregnancy. Due to sexual dimorphism, we did not assign age class to sperm whales (*Physeter macrocephalus*) of unknown gender between 900 and 1500 cm and excluded these records from age-related analyses.

To integrate variable uses and interpretations of cause of death, we accepted the most likely scenario as the presumed cause of death. We used histopathological and gross necropsy evidence to determine whether human interaction was a causal factor of death (Geraci & Lounsbury 1989). We categorized living whales as having a serious injury if the injury would “likely result in mortality” (NOAA 1999) attributable to either entanglement or vessel strike on the basis of an additional set of criteria (e.g., fishing line constricted any body part or was likely to become constricting as the whale grew) (Glass et al. 2010). Thus, we considered a serious injury (*n* = 117) a mortality. We categorized cause of death as entanglement, vessel strike, other human cause (gunshot wounds, marine debris), nonhuman cause (complications at birth, parasitic infection, live stranding), and undetermined (due to decomposition, an inaccessible carcass, or where no necropsy data were provided to indicate cause of death).

We identified duplicate records and consolidated relevant information from each contributing source to produce one complete record. When we found discrepancies (e.g., mismatched dates of sighting) in overlapping data sets, we consulted original reports. We removed reports of live animals successfully disentangled or returned to sea when these events did not qualify as serious injuries.

### Statistical Analyses

Potential biological removal (PBR) is a measure of sustainable human-caused mortality to a species (Wade 2008) and is calculated as


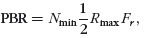
(1)

where *R*_max_ is the maximum theoretical or estimated net productivity rate, *F_r_* is a recovery factor between 0.1 and 1.0, and *N*_min_, the minimum population estimate, which is calculated as


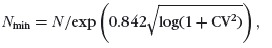
(2)

where the population estimate *N* has a corresponding coefficient of variation (CV). Since 1995, PBR has been used to calculate the species-specific level below which human-induced mortality must be reduced. By definition, PBR also provides a measure of population abundance. To assess the sustainability of the level of human-caused mortality to populations of large whales, we followed a method similar to that of US federal agencies (e.g., Glass et al. 2010) in which we compared the number of human-caused mortalities (cause of death entanglement, vessel strike, and other human cause) per species per year with the calculated species-specific PBR for those years (NMFS 2012). We applied this method for years following the initial calculation of PBR for all stocks in 1995 (2000 for Gulf of Maine humpback whales due to management unit redefinition). We excluded blue (*Balaenoptera musculus*) and sei (*Balaenoptera borealis*) whales because PBR was poorly and inconsistently established for these species over the study period due to insufficient stock-assessment data. We included mortalities in US and Canadian waters because the population ranges over which abundance estimates are calculated span US and Canadian waters. As is assumed for similar comparisons of human-caused mortalities to PBR levels conducted by federal agencies (Glass et al. 2010), we assumed all humpback events occurring in or near US and southeast Canadian waters involved the Gulf of Maine stock.

We used chi-square tests to determine departures from expected mortality events for sex, age class, and cause of death and 2-way contingency tables and associated Pearson's chi-square tests to assess changes in the expected proportions of mortalities observed in US and Canadian waters between 2 nonoverlapping periods (representing different levels of mitigation effort): 1 April 1990–31 March 2003 and 1 April 2003–31 December 2009. We chose the month of April to reflect seasonality in the geographical distribution of whale populations as a whole and the year 2003 because numerous mitigation actions were taken in 2002 to address concerns about mortality of large whales from entanglement and vessel strikes (Supporting Information).

We categorized carcasses as stranded or floating to reduce the effect of variable effort in at-sea carcass detection. We defined mortality events within 5.56 km (equal to 3 nautical miles, i.e., state waters of the United States) of the smoothed coastline as stranded (*n* = 894) because these carcasses were most likely to land within their specific geographic location and events farther offshore as floating (*n* = 868) because their coastal geographic location could not be similarly assumed or used in coastal distribution analyses. We calculated the coordinates of each stranded event in ArcGIS 9.2 (ESRI 1999) as a distance along the coastline from the U.S.-Mexico border (26.0°N) to Escuminac, New Brunswick (47.05°N), and smoothed embayments 24.6 km across or less between the north and south points of entrance to reduce coastline complexity.

To assess the significance of changes in the geographical distribution of mortality following extensive management changes, reflected in the location along the coast of stranded cases, we used a Cramér-von Mises test with randomization (Zhang & Wu 2011). We used linear regression on the proportion of cases where necropsies had been performed and for which cause of death had been determined to identify changes in effort over the study period.

To address changes in the rate of mortalities attributed to specific causes, we used maximum likelihood to fit a multinomial logistic model. For reference, subscripts refer to *e*, entanglement; *v*, vessel strike; *o*, other known causes; *s*, stranded; *f*, floating. **N**_**s**_ **(t)** = [*N_es_*(*t*), *N_vs_*(*t*), *N_os_*(*t*)] was the vector of counts of stranded events in year *t* attributable to entanglement, vessel strike, and other known causes (other and nonhuman). The corresponding total number of stranded events (*M_s_*) with an attributable cause of death was *M_s_*(*t*) = *N_es_*(*t*) + *N_vs_*(*t*) + *N_os_*(*t*). Similarly, **N**_**f**_ **(t)** = [*N_ef_*(*t*), *N_vf_*(*t*), *N_of_*(*t*)] was the vector of counts of floating events in year *t* with total count (*M_f_*) equal to *M_f_(t*) = *N_ef_*(*t*) + *N_vf_*(*t*) + *N_of_*(*t*). The basic statistical model was that, conditional on the observed value *m_s_*(*t*) of *M_s_*(*t*), *N_s_*(*t*) had a multinomial distribution with *m_s_*(*t*) trials and probability vector for stranded events (**p**_**s**_) equal to 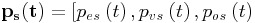. Similarly, we assumed, conditional on the observed value *m_f_*(*t*) of *M_f_*(*t*), that *N_f_*(*t*) had a multinomial distribution with *m_f_*(*t*) trials and probability vector for floating events (**p**_**f**_) equal to **p**_**f**_ **(t)** = [*p_ef_*(*t*), *p_vf_*(*t*), *p_of_*(*t*)].

To test for a trend over time in probability vectors for stranded and floating events, *p_s_(t)* and *p_f_(t*) respectively, we adopted a multinomial logistic model (Hosmer & Lemeshow 2005) that allowed for a linear trend in time:


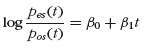
(3)

and


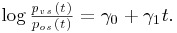
(4)

With inverse transformation


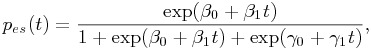
(5)


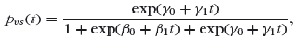
(6)

and


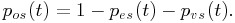
(7)

Although we could not assume that *p_s_*(*t*) = *p_f_*(*t*), we assumed the trend parameters (β_1_ and γ_1_) in the 2 data sets were the same, so that


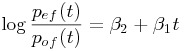
(8)

and


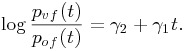
(9)

We used likelihood ratio to test the null hypothesis (β_1_ = γ_1_ = 0) of no change over time in mortality rates against the general alternative hypothesis.

## Results

### Mortality Events

**Figure 1:**
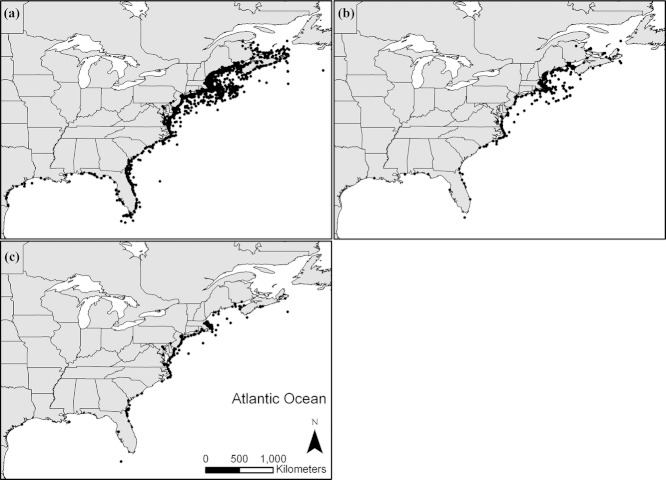
Locations of all (a) observed mortalities and serious injuries of large whales in the Northwest Atlantic and Gulf of Mexico from 1970 through 2009 and locations of mortalities attributed to (b) entanglement and (c) vessel strike. Events located inland occur in river inlets.

Over 40 years (1970–2009 inclusive), at least 1762 confirmed mortalities involved 8 species of large whales (Fig. 1a). Fourteen mortalities (0.8%) involved 2 or more individuals (maximum 12 individuals). These cases of multiple mortalities included a mass stranding of 11 sperm whales (1980), a presumed saxitoxin poisoning event of humpbacks in 1987 (Geraci et al. 2001), and a multispecies event in 2003 (Cassoff et al. 2008).

Individuals were identified to species in 1472 (85%) cases. Mortality reports involved all species of large whales found in the Northwest Atlantic except the bowhead whale (*Balaena mysticetus*) (Table 2). Cause of death was determined for 750 individuals (42.6%) (Table 2), 66.9% of which resulted from interaction with human activities. Necropsies were performed on 478 individuals (27.1%); cause of death was determined for 74.3% of these cases. The leading cause of death for all species combined was entanglement in fishing gear (*n* = 323) (Fig. 1b), followed by nonhuman causes (*n* = 248) and vessel strikes (*n* = 171) (Fig. 1c). Entanglement was the leading presumed cause of death for humpback, minke (*Balaenoptera acutorostrata*), and unidentified whales; vessel strike was the leading cause for fin and right whales; and nonhuman causes were the leading cause for sperm whales (χ^2^ > 22.9, *p* < 0.001 for all). Although not statistically significant, sei whales died more often than expected by vessel strike, and Bryde's (*Balaenoptera edeni*) whales died more often than expected due to nonhuman causes (Table 2) (χ^2^ = 8.94, *p* = 0.063; χ^2^ = 2.08, *p* = 0.721, respectively).

**Table 2:** Total number and percentage of mortalities per species and cause of death from 1970–2009 (inclusive), average annual potential biological removal (PBR), average difference between PBR and the number of human-caused mortalities per year, and average percent PBR met or exceeded by human-caused mortalities per year

*Cause of death*	*Blue whale*	*Bryde's whale*	*Fin whale*	*Humpback whale*	*Minke whale*	*Right whale*	*Sei whale*	*Sperm whale*	*Unknown*	*Total (%)*
EN	0	2	26	116	101	31	5	9	33	323 (18.33)
VS	1	1	59	31	17	38	9	6	9	171 (9.7)
ENVS	0	0	0	4	0	1	0	0	0	5 (0.28)
OH	0	0	1	0	1	0	0	0	1	3 (0.17)
NH	1	5	30	52	57	17	3	76	7	248 (14.07)
UN	2	7	141	270	220	35	12	85	240	1012 (57.43)
Total (%)	4 (0.23)	15 (0.85)	257 (14.59)	473 (26.84)	396 (22.47)	122 (6.92)	29 (1.65)	176 (9.99)	290 (16.46)	1762
Average (SD) PBR/year 1995–2009	NA^b^	0.2 (0.1)	3.9 (0.6)	1.4 (0.7)	26.0 (6.8)	0.1 (0.2)	NA	7.0 (2.8)	NA	
Average (SD) difference from PBR/year 1995–2009	NA	−0.1 (0.4)	−0.5 (1.8)	6.7 (3.5)^c^	−21.5 (6.9)	3.1 (0.5)	NA	−6.6 (3.0)	NA	
Average (SD) % PBR met or exceeded/year	NA	88.9 (2.67)	90.2 (46.0)	579 (284)	18.3 (9.4)	650 (379)	NA	8.4 (11)	NA	

a EN, entanglement; VS, vessel strike; ENVS, entanglement and vessel strike; OH, other human cause; NH, nonhuman cause; UN, undetermined; see text for definition of categories.

b Not applicable.

c Calculated only for years 2000–2009 due to management unit redefinition.

**Figure 2:**
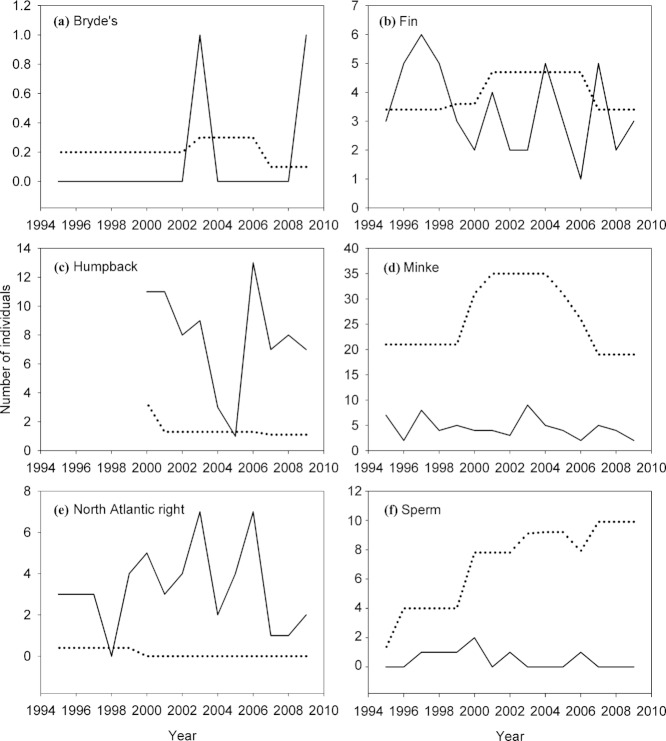
Annual potential biological removal (PBR), a species-specific measure of sustainable human-caused mortality (dotted line) and human-induced (HI) mortality (solid line) for (a) Bryde's, (b) fin, (c) humpback, (d) minke, (e) right, and (f) sperm whales.

Over the study period, 473 humpback whales (the greatest proportion, 32%) and 122 right whales died. Human-caused mortalities of these species exceeded their PBRs by an average of 6.4 (SD 3.5) and 3.1 (0.5) individuals/year (579% and 650%), respectively (Table 2 & Fig. 2). For these 2 species, PBR was exceeded in all years except one. Entanglement mortalities alone exceeded PBR for humpback and right whales by an average of 4.6 (SD 2.7) and 1.5 (1.4) individuals/year (430% and 300%), respectively. On average the annual observed human-caused mortality of Bryde's, minke, fin, and sperm whales was below PBR.

Sex, which was determined for 783 individuals (44.4%) (Supporting Information), differed from parity only for minke whales (χ^2^ = 6.49, *p* = 0.011) for which there was significantly higher female mortality. Significantly higher female mortality also occurred in right whales (total mortality: 46 females vs. 39 males - χ^2^ = 5.12, *p =* 0.024; entanglement, vessel-strike, other human causes: *n* = 32 females vs. 19 males - χ^2^ = 9.01 *p* = 0.003) (population 58% male [NARWC 2011]).

Age class (calf, subadult, adult) was estimated for 1164 individuals (66.1%). Subadults had the highest mortality (*n* = 602) across all causes of death, followed by calves (*n* = 346) and then adults (*n* = 216). Adult females and subadult males had significantly greater mortality than adult males and subadult females in humpback and sperm whales (χ^2^ = 7.83, 27.3; *p* = 0.020, < 0.001, respectively). Right whales had significantly higher male calf mortality and female subadult and adult mortality than female calves and subadult males (χ^2^ = 7.28, *p* = 0.026). Cause of death differed significantly across age classes (χ^2^ = 29.5, *p* < 0.001). Calves died significantly more often from non-human causes (*n* = 98), subadults significantly more often by vessel strike (*n* = 97), and adults significantly more often by entanglement (*n* = 35) than expected (Supporting Information).

Pregnancy was detected in 13 females: 5 minke, 3 right, 2 fin, 2 sperm, and 1 sei. Cause of death was determined for 8 of these animals. Vessel strike was the leading cause (*n* = 4), followed by entanglement (*n* = 2) and nonhuman causes (*n* = 2; 1 presumed saxitoxin, 1 mass-stranding).

### Temporal and Spatial Trends

**Figure 3:**
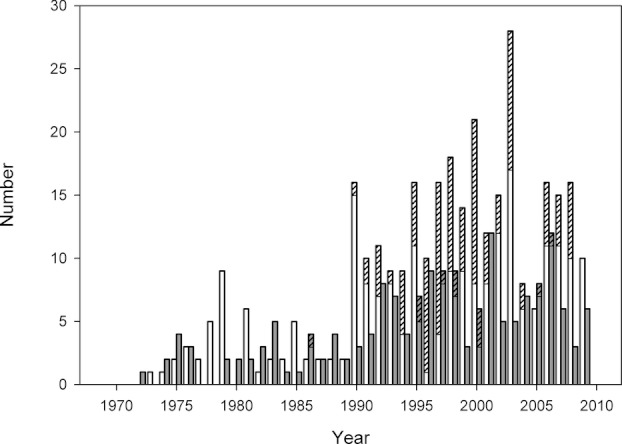
Annual number of mortalities of large whales (no hatching) and serious injuries (hatched lines) attributed to vessel strike (shaded) and entanglement (unshaded).

Maximum likelihood estimates were 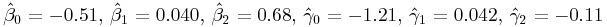. Positive slope parameters (β_1_ and γ_1_) indicated increasing probability vectors for entanglement and vessel-strike mortalities over time for both stranded and floating carcasses (Fig. 3).

**Figure 4:**
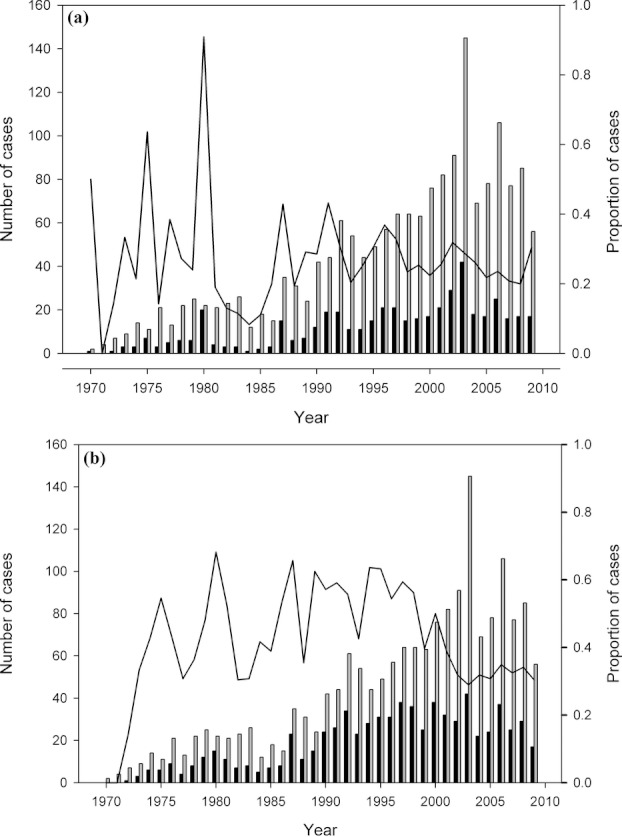
(a,b) Number and proportion of annual mortalities of large whales (grey bars); (a) number and proportion of cases for which necropsies were performed (black bars) and the resulting annual proportion of necropsied cases (black line); and (b) number and proportion of cases in which cause of death was determined (black bars) and the resulting annual proportion of determined cause of death (black line).

The proportion of cases for which necropsies were performed decreased slightly since 1970 (*p* = 0.024, *R*^2^ = 0.007) (Fig. 4a). On average from 1990–2009 necropsies were performed on 27.4% of cases per year (SD 5.96). The proportion of cases for which cause of death was determined linearly increased (*p* = 0.026, *R*^2^ = 0.030; Fig. 4b) from 1970 to 2000, after which it decreased.

### Spatial Trends in Mortality

**Figure 5:**
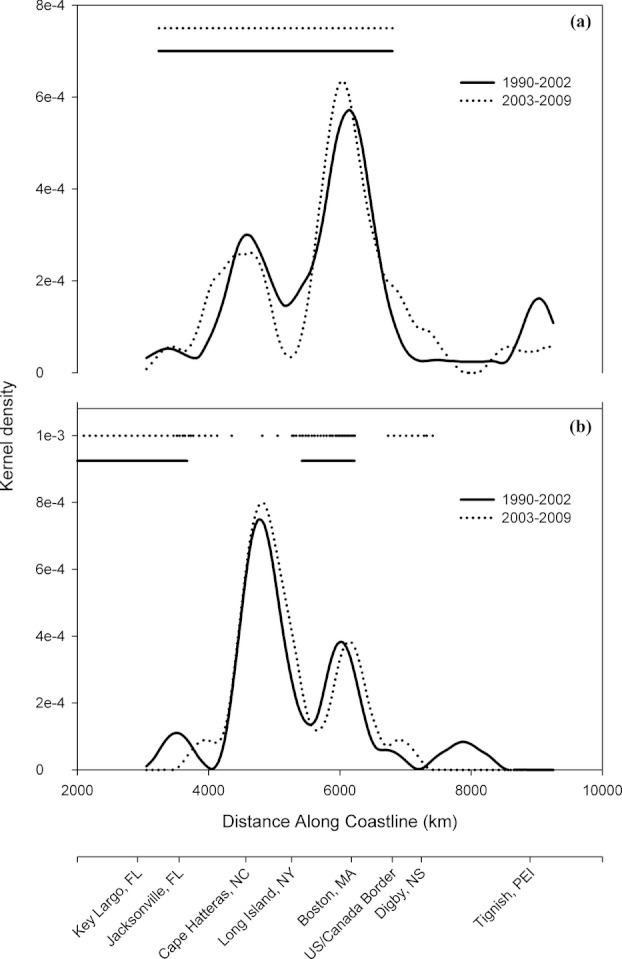
Smoothed-kernel density estimates of the distance along the coastline of mortality events for stranded large whales due to (a) entanglement and (b) vessel strike from 1990–2002 and 2003–2009, and (horizontal lines at the top of each graph) spatial extent of efforts implemented to address the specific sources of mortality in each period (FL, Florida; NC, North Carolina; NY, New York; MA, Massachusetts; NS, Nova Scotia; PEI, Prince Edward Island).

Events were not uniformly distributed. Most mortalities occurred in particular regions (Fig. 1). Smoothed-kernel estimates of mortality density for 1990–2002 and 2003–2009 suggested relative increases in mortality density in South Carolina, Virginia, Massachusetts, and Nova Scotia and relative decreases off New York and Prince Edward Island (Fig. 5). Cramér-von Mises tests indicated no significant differences in the spatial distribution of all stranded mortalities (*p* = 0.187) or of vessel strike mortalities (*p* = 0.937) (Fig. 5b) in the United States and Canada before and after 2003; however, entanglement mortalities differed significantly in their distribution before and after 2003 (*p* = 0.010) (Fig. 5a). Proportions of stranded and floating mortalities in US and Canadian waters before and after 2003 do not differ significantly from expected proportions for all causes of death (χ^2^ = 3.15, *p* = 0.076) or for determined entanglement (χ^2^ = 594, *p* = 0.441) and vessel-strike (χ^2^ = 0.004, *p* = 0.427) mortalities.

## Discussion

Our results show significantly increasing trends in large whale mortality probabilities associated with entanglement and vessel strikes despite regulatory efforts to reduce these risks. We found 66.9% of mortalities were related to human activities, which consistently exceeds established PBR for right and humpback whales. So far, regulatory efforts have been insufficient to reduce the lethal effects of human activities on large whales on a population-range basis, although we cannot exclude the possibility of success of targeted measures for specific localized areas that were not within the resolution of our analyses. In interpreting the causal factors of these trends, changes in industry effort; fluctuations in population sizes of large whales; changes in effort in stranding response, mortality detection, and large whale forensics; and compliance with regulations must be considered.

Factors other than regulation may affect shipping patterns and densities and may have acted as confounding factors in our analyses. For instance, the economic downturn beginning in late 2007 reduced consumer demand for goods transported by ship and thus the amount of cargo distributed, although levels of domestic shipping remained fairly constant (P. Turner, personal communication). The complexity of the shipping industry makes it difficult to resolve a simple metric of economic effects on spatial and temporal shipping trends, and changes in fishing effort over these spatial and temporal scales are too varied to determine a useable value.

Our results provide minimum estimates of mortality events. Mortality events may have gone undetected or have been insufficiently documented (i.e., no cause of death determined). Despite increased awareness, collaboration, and allocation of resources toward carcass detection and retrieval, a substantial proportion of mortalities are not examined or observed (range: 0–6.2% for Gulf of Mexico species [Williams et al. 1998] to 17–33% for extensively studied right whales [Knowlton & Kraus 2002; Kraus et al. 2001]).

The development of stranding programs and the consistency and quality of event documentation varied over the years of the study. Systematic data collection from events in the United States began in 1972 when the Marine Mammal Protection Act was enacted; response networks in Canada developed more recently (around 1990) (Nemiroff et al. 1994). Standard protocols, diagnostic gross necropsies, and collection of samples for histopathology, microbiology, genetics, and biotoxin analyses (McLellan et al. 2000; Campbell-Malone et al. 2008) have increased.

Variable detection effort inshore versus offshore supports our separation of data into subsets of stranded and floating carcasses. Stranded large whales are unlikely to escape notice and have a higher degree of reporting due to relatively constant public and media interest. Detection of floating carcasses has increased, due to opportunistic reporting by vessel operators and to recent coordination between dedicated surveys and fishery, coast guard and naval agencies. The interest in towing carcasses ashore or to examine them at sea has also increased (beginning in 1986). The proportion of necropsies performed remained relatively stable even though the number of mortalities increased (Fig. 4a). The proportion of cases for which cause of death was determined increased since 1970 (Fig. 4b), although a recent decrease (since 2000) can be attributed to increased detection of floating or inaccessible carcasses to which a cause of death cannot be assigned. We believe carcass examination to determine the cause of death is crucial to identifying risk factors and management successes. Where possible, a system-wide standard process to acquire data from all species, not just right whales, should exist to increase determination of cause of death.

In cases for which cause of death was determined, human activities were associated with 66.9% of large-whale mortalities; entanglement was the leading cause. Primary cause of death may reflect the relative vulnerability of certain species to specific industries. Fin and right whales are more often struck by vessels (Vanderlaan & Taggart 2004; Van Waerebeek & Leaper 2008), humpback and minke whales are more often fatally entangled (Volgenau et al. 2012; Song et al. 2009), and sperm whale mortalities are primarily natural and associated with live stranding (Lucas & Hooker 2001). We suggest cause-of-death determinations we examined were biased toward entanglement and nonhuman factors due to the cryptic nature of blunt trauma associated with vessel-strike mortalities.

Entanglement occurs more frequently in juveniles (Lien 2011; Knowlton & Kraus 2002). Adults do become entangled, but it is likely that dying is protracted: individuals may become seriously entangled as subadults and succumb to entanglement injuries as adults. In contrast, death from vessel strikes is relatively instantaneous. Furthermore, detection rates may vary by size or age class; smaller or subadult whales may lack the force required to dislodge gear and may therefore remain anchored at depth (Lien 2011; Cassoff et al. 2008).

High mortality of adult females and pregnant individuals is problematic for slow-growing and recovering populations. For right whales, the prevention of 2 female mortalities per year has the potential to increase population growth to replacement levels (Fujiwara & Caswell 2011). Although this statistic is less dramatic for other large whales, the lost lifetime reproductive potential for any endangered, k-selected species is likely to be severe. Because the recovery factor included in PBR calculations can be adjusted to reflect higher-than-expected female mortality, we suggest this adjustment be made for minke and right whales.

Results of the comparisons of mortalities with established PBR levels (Fig. 2) indicated the sustainability of the level of human-caused mortality to specific populations of large whales. Since 1995 and 2000, respectively, average human-caused mortalities of right and humpback whales exceeded their PBRs, whereas Bryde's, fin, minke, and sperm-whale mortalities were below PBR. This difference likely reflects the high proportion of natural mortality in sperm whales, the size of the minke whale population relative to this species’ rate of detected entanglement, and the lack of basic population data for Bryde's whales. The goal of the Atlantic Large Whale Take Reduction Plan (NOAA 2011) is to reduce mortalities and serious injuries related to commercial fishing to below PBR within 6 months of its implementation; that goal has been met for only 1 year (and by a fraction of an individual) for humpback or right whales since its establishment (NOAA 2010). We suggest basic population data be collected at least every 5 years and that regular data collection begin on blue, Bryde's, and sei whales, where it is almost completely lacking.

Consistency in the spatial distribution of stranded carcasses is due to presence of high-risk areas and geographical factors (Fig. 5). Compared with the spatial extent of regulations, vessel-strike mortality continues to be highest in the mid-Atlantic coast. We suggest efforts in this region be aimed at reducing mortality risk.

In 2003 efforts designed to address large-whale mortality became more frequent and extensive (Supporting Information). Since then policies have continued to evolve, and their effects are manifest in the years 2003–2009. Although some regulations have been evaluated for their potential to reduce the rates and relative risk of lethal vessel strikes in a given region (e.g., Vanderlaan et al. 2007), the effects of these regulations are unlikely to be detected over large areas because they have only been in place for ≤6 years (Pace 2011).

A U.S. regulation (Ship Strike Rule, effective in 2008 [Table S1]) designed “to reduce or eliminate the threat of vessel-strikes” to right whales expires in December 2013 (NOAA 2005). No significant decrease in the frequency of vessel-strike mortalities is yet apparent (Pace 2011). Rule compliance in the first year was poor; only 20% of transiting vessels fully complied with the ≤18.5 km/h (i.e., 10 knots) limit (MMC 2010). Regional estimates indicate greater compliance off Massachusetts (approximately 50%) (MMC 2010) and in the southeastern United States calving ground (75%) (Lagueux et al. 2005). As with mandatory speed restrictions, recommendations put in place since 2003 on vessel speeds and routing have not had a measurable effect on reducing the number of lethal vessel strikes on the scale of our analyses for 8 large-whale species. However, to better assess the efficacy of the Ship Strike Rule prior to expiration or renewal, the current analyses should be repeated early in 2013 and mortalities before and after 2008 should be compared.

It is premature to conclude the 2008 Ship Strike Rule has had any direct effects on reducing lethal vessel strikes to right whales or other large whale species. The rule is up for review in 2013, and we suggest the review focus on assessing overall compliance, redesigning regulation and increasing awareness and monitoring to address low compliance in particular areas, considering actions that benefit all species of large whales over larger areas (e.g., Schick et al. 2006), and addressing the high mortality in the mid-Atlantic United States, where only limited efforts have been taken to reduce vessel strikes. Lagueux et al. (2005) describe greater compliance in the southeastern United States to recommended vessel routing compared with mandatory speed restrictions. This difference may indicate practical considerations of the shipping industry. Greater success in reducing vessel-strike mortalities may be achieved by restructuring lanes than by restricting speeds.

We present analyses crucial to evaluating conservation decisions and that can provide managers with direction for modifying current methods. Although our analyses have specific relevance to large whales in the Northwest Atlantic, they are equally applicable to mortality-driven conservation issues for other populations of large whales (Panigada et al. 2008), elephants (Sarma et al. 2006), tigers (Kerley et al. 2008), and amphibians (Glista et al. 2006). The continued detection and investigation of relatively rare events and their inclusion in time series of data are crucial to the evaluation of the efficacy of existing management of living ocean resources and to improve rules that address factors associated with unsustainable levels of mortality in large whales.

## Supplementary material

Disclaimer: Supplementary materials have been peer-reviewed but not copyedited.
