# Supplementary material for: Assessment of Management to Mitigate Anthropogenic Effects on Large Whales
Source: Conserv Biol. 2012 Oct 1;27(1):121–33. doi: 10.1111/j.1523-1739.2012.01934.x (PMC3562480; doi:10.1111/j.1523-1739.2012.01934.x)
Supplement: Supplementary file 1 [file cobi0027-0121-SD1.pdf]

Appendix S1.

Timeline of major mitigation efforts at state, Canadian/US Federal and international levels to increase awareness and reduce the potential for human-associated mortality to large whale species.

Table S1. Timeline of major mitigation efforts at state, Canadian/US Federal and international levels to increase awareness and reduce the potential for human-associated mortality to large whale species.

Event	Goal	Date or annual time period of implementation	Location	Reference
Establishment of right whale conservation areas	Promotes awareness and education among mariners of high concentrations of right whales.	1993	Grand Manan Basin and Roseway Basin, Canada	(Brown et al. 1995)
Establishment of right whale critical habitat	Ensures federal agency actions will not destroy or adversely modify critical habitat.	5 July 1994	Cape Cod Bay (CCB), Stellwagen Bank, Great South Channel (GSC), coastal waters of Georgia and east coast of Florida	(59 FR 28805; NOAA 1994)
Approach restrictions	Restricts vessels and aircraft from approaching within 460m (500 yards) of right whales to reduce the possibility of interaction and injury.	17 March 1997	All US waters	(62 FR 6729; NOAA 1997)
Implementation of Atlantic Large Whale Take Reduction Plan (ALWTRP)	Seeks to reduce serious injury and mortality to right, humpback, fin, and minke whales.	1 April 1999	US Atlantic Exclusive Economic Zone (EEZ) from Maine (43.9667ºN) through Florida (26.775ºN).	(64 FR 7529; NOAA 1999)
Mandatory Ship Reporting Systems	Reduce vessel-whale collisions by providing timely information to mariners entering areas used by right whales.	As of 1 July 1999; Year-round (Northeast; NE); 15 November – 16 April (Southeast; SE)	Massachusetts coastal waters (NE); Georgia and Florida coastal waters (SE).	(Silber et al. 2002)

Table S1. Timeline of major mitigation efforts at state, Canadian/US Federal and international levels to increase awareness and reduce the potential for human-associated mortality to large whale species.

Event	Goal	Date or annual time period of implementation	Location	Reference
Gear Modifications	Requires buoy weak line links, net panel weak links with anchoring systems, gear marking; restricts number of buoy lines.	22 January 2001	Northern Inshore Lobster Waters, CCB Restricted Area, GSC Restricted Lobster Area, Northern Nearshore Lobster Waters Area, Southern Nearshore Lobster Waters, Offshore Lobster Waters	(65 FR 80368; NOAA 2000)
Dynamic Area Management (DAM) scheme implemented	Restricts use of lobster trap/pot and gillnet fishing gear to protect aggregations of right whales outside of critical habitat.	8 February 2002	US waters North of 40°N. DAM zones: areas triggered by aggregations of 3 or more right whales outside of previously established management areas or critical habitat zones, or within and outside these areas when seasonal management is not in effect.	(67 FR 1133; NOAA 2002a)
Gear Modifications	Replaces existing Gillnet Take Reduction Technology List with mandatory weak link requirements.<br>Allows the use of neutrally buoyant line in lobster fishing gear.	11 February 2002	All ALWTRP Regulated Lobster Waters and ALWTRP Regulated Gillnet Waters.	(67 FR 1300; NOAA 2002b)

Table S1. Timeline of major mitigation efforts at state, Canadian/US Federal and international levels to increase awareness and reduce the potential for human-associated mortality to large whale species.

Event	Goal	Date or annual time period of implementation	Location	Reference
Seasonal Area Management (SAM) scheme implemented	Prohibits use of floating ground line and buoy line; establishes the number, strength, location of weak links; limits single buoy line per net string.	As of 1 March 2002; SAM West 1 March – 30 April, SAM East 1 May – 31 July.	Massachusetts coastal waters.	(67 FR 1142; NOAA 2002c)
Southeast US (SEUS) Gillnet Prohibition	Prohibits straight set gillnets during nighttime hours.	As of 2002; 15 November – 31 March, annually.	Coastal waters of Georgia and east coast of Florida	(67 FR 59471; NOAA 2002d)
Bay of Fundy Traffic Separation Scheme (TSS) Amendment	Alters vessel traffic around highest concentration of right whales to reduce possibility of vessel-collisions; International Maritime Organization (IMO) adopted.	1 July 2003	Bay of Fundy, Canada	(IMO 2003)
DAM Gear Modifications	Allows use of specific anchored gillnet and lobster trap/pot modifications that reduce entanglement risk	25 September 2003	DAM zones, as above.	(68 FR 51195; NOAA 2003)
Recommended Voluntary Ship Traffic Routes	Suggests alternate ship traffic routes to mariners to reduce vessel activity in areas frequented by whales.	30 November 2006	Jacksonville and Fernandina, FL; Brunswick, GA; Cape Cod Bay, MA.	(NOAA 2006)

Table S1. Timeline of major mitigation efforts at state, Canadian/US Federal and international levels to increase awareness and reduce the potential for human-associated mortality to large whale species.

Event	Goal	Date or annual time period of implementation	Location	Reference
Boston TSS Amendment	Shifts traffic pattern to avoid large aggregations of whales; IMO adopted.	1 July 2007	Boston, Cape Cod Bay, MA waters.	(IMO 2006)
Changes to Boundaries and Seasons, Gear Modifications	Extends ALWTRP gear modifications for regulated areas to the eastern edge of the EEZ; requires weak links of appropriate breaking strength; replaced/eliminated SAM & DAM programs.	5 April 2008; North of 40°00N year-round; between 32°00N and 40°00N 1 September – 31May; between 29°00N and 32°00N 15 November – 15 April; between 27°51N and 29°00N 1 December – 31	All ALWTRP-Regulated Trap/Pot Waters	(72 FR 57104; NOAA 2007a)
Roseway Basin Area To Be Avoided	Recommendatory, seasonal; limits transits of vessels (>300 gross tonnage) at peak time of abundance in right whale critical habitat. IMO adopted.	As of 2008; 1 June – 31 December annually.	Roseway Basin, Southwest Scotian Shelf, Canada.	(IMO 2007)

Table S1. Timeline of major mitigation efforts at state, Canadian/US Federal and international levels to increase awareness and reduce the potential for human-associated mortality to large whale species.

Event	Goal	Date or annual time period of implementation	Location	Reference
Speed Restrictions	Restricts vessel speeds to no more than 10 knots, to reduce likelihood of death in the event of a vessel-whale collision.	9 December 2008 – 9 December 2013:<br><br>SEUS 15 November – 15 April; Mid-Atlantic 1 November – 3 April; CCB 1 January – 15 May; Off Race Point (ORP) 1 March – 30 April; GSC 1 April – 31 July.	SEUS: St. Augustine, FL to Brunswick, GA.<br><br>Mid-Atlantic: Brunswick, GA to Rhode Island; Ports of New York, New Jersey, Philadelphia PA, Wilmington VA, Moorehead City NC and Beaufort NC; Entrance of Chesapeake Bay; Block Island Sound<br>NEUS: CCB, ORP, GSC<br><br>Any Triggered DMA zones (as above).	(73 FR 60173; NOAA 2008a)
Broad-Based Sinking Groundline Requirement	Implements broad-based sinking groundline requirement for all trap/pot fisheries; eliminates DAM fishing restrictions.	5 April 2009	All ALWTRP-Regulated Trap/Pot Waters	(72 FR 57104, 73 FR 19171, 73 FR 51228; NOAA 2007a, 2007b, 2008b)
Great South Channel Area To Be Avoided	Voluntary, seasonal; limits vessel transits at peak time of abundance in right whale critical habitat. IMO adopted.	1 June 2009; Annually 1 April – 31 July	Great South Channel, Massachusetts.	(IMO 2008a)

Table S1. Timeline of major mitigation efforts at state, Canadian/US Federal and international levels to increase awareness and reduce the potential for human-associated mortality to large whale species.

Event	Goal	Date or annual time period of implementation	Location	Reference
Modification of Boston TSS	Modifies existing lanes to reduce the threat of collisions; IMO adopted.	1 June 2009	Massachusetts.	(IMO 2008b)
Designation of critical habitat for right whales in the Grand Manan Basin and Roseway Basin.	Establishes critical habitat areas federally protected under the Species At Risk Act (SARA).	1 June 2009	Bay of Fundy and Roseway Basin, Canada	(Brown et al. 2009)

Literature Cited

Brown, M.W., J.M. Allen, and S.D. Kraus. 1995 The designation of seasonal right whale conservation areas in the waters of Atlantic Canada. Pages 90-98 in N.L. Shackell and J.H.M. Willison, editors. Marine protected areas and sustainable fisheries. Science and Management of Protected Areas Association, Wolfville, Nova Scotia.

Brown, M.W., D. Fenton, K. Smedbol, C. Merriman, K. Robichaud-Leblanc, and J.D. Conway. 2009. Recovery strategy for the North Atlantic right whale (*Eubalaena glacialis*) in Atlantic Canadian waters. Species at Risk Act Recovery Strategy Series. Fisheries and Oceans Canada, Ottawa, Ontario.

IMO (International Maritime Organization). 2003. New and amended traffic separation schemes. Ref T2/2.07, COLREG.2/Circ.52. IMO, London.

IMO (International Maritime Organization). 2006. New and amended traffic separation schemes. Ref T2-OSS/2.7.1, COLREG.2/Circ.58. IMO, London.

IMO (International Maritime Organization). 2007. Routeing measures other than traffic separation schemes. Ref. T2-OSS/2.7, SN.a/Circ.263. IMO, London.

IMO (International Maritime Organization). 2008a. Routeing measures other than traffic separation schemes. Ref. T2-OSS/2.7.1, SN.1/Circ.272, IMO, London.

IMO (International Maritime Organization). 2008b. Routeing of ships, ship reporting, and related matters. Amendment to the Traffic Separation Scheme "In the Approach to Boston, Massachusetts". Ref. NAV 54/3/XX, IMO, London.

NOAA (National Oceanic and Atmospheric Association). 1994. Designated Critical Habitat; Northern Right Whale. Federal Register **59**:28805-28834.

NOAA (National Oceanic and Atmospheric Association). 1997. North Atlantic Right Whale Protection. Federal Register **62**:6729-6738.

NOAA (National Oceanic and Atmospheric Association). 1999. Taking of Marine Mammals Incidental to Commercial Fishing Operations; Atlantic Large Whale Take Reduction Plan Regulations. Federal Register **64**:7529-7556.

NOAA (National Oceanic and Atmospheric Association). 2000. Taking of Marine Mammals Incidental to Commercial Fishing Operations; Atlantic Large Whale Take Reduction Plan Regulations. Federal Register **65**:80368-80381.

NOAA (National Oceanic and Atmospheric Association). 2002a. Taking of Marine Mammals Incidental to Commercial Fishing Operations; Atlantic Large Whale Take Reduction Plan Regulations. Federal Register **67**:1133-1142.

NOAA (National Oceanic and Atmospheric Association). 2002b. Taking of Marine Mammals Incidental to Commercial Fishing Operations; Atlantic Large Whale Take Reduction Plan Regulations. Federal Register **67**:1300-1314.

NOAA (National Oceanic and Atmospheric Association). 2002c. Taking of Marine Mammals Incidental to Commercial Fishing Operations; Atlantic Large Whale Take Reduction Plan Regulations. Federal Register **67**:1142-1160.

NOAA (National Oceanic and Atmospheric Association). 2002d. Taking of Marine Mammals Incidental to Commercial Fishing Operations; Atlantic Large Whale Take Reduction Plan Regulations. Federal Register **67**:59471-59477.

NOAA (National Oceanic and Atmospheric Association). 2003. Taking of Marine Mammals Incidental to Commercial Fishing Operations; Atlantic Large Whale Take Reduction Plan Regulations. Federal Register **68**:51195-51201.

NOAA (National Oceanic and Atmospheric Association). 2006. News From NOAA For Immediate Release, November 17, 2006.

NOAA (National Oceanic and Atmospheric Association). 2007a. Taking of Marine Mammals Incidental to Commercial Fishing Operations; Atlantic Large Whale Take Reduction Plan Regulations. Federal Register **72**:57104-57194.

NOAA (National Oceanic and Atmospheric Association). 2007b. Taking of Marine Mammals Incidental to Commercial Fishing Operations; Atlantic Large Whale Take Reduction Plan Regulations; Correction. Federal Register **73**:19171.

NOAA (National Oceanic and Atmospheric Association). 2008a. Endangered Fish and Wildlife; Final Rule To Implement Speed Restrictions to Reduce the Threat of Ship Collisions With North Atlantic Right Whales. Federal Register **73**:60173-60191.

NOAA (National Oceanic and Atmospheric Association). 2008b. Taking of Marine Mammals Incidental to Commercial Fishing Operations; Atlantic Large Whale Take Reduction Plan Regulations. Federal Register **73**:51228-51242.

Silber, G.K., L.I. Ward, R. Clarke, K.L. Schumacher, and A.J. Smith. 2002. Ship traffic patterns in right whale critical habitat: Year one of the Mandatory Ship Reporting System. NOAA Tech Memo NMFS OPR 20, 27 pp.
