# Supplementary material for: Assessment of Management to Mitigate Anthropogenic Effects on Large Whales
Source: Conserv Biol. 2012 Oct 1;27(1):121–33. doi: 10.1111/j.1523-1739.2012.01934.x (PMC3562480; doi:10.1111/j.1523-1739.2012.01934.x)
Supplement: Supplementary file 2 [file cobi0027-0121-SD2.pdf]

Table S2. Determined male and female mortality for 8 large whale species in the coastal Northeast Atlantic, 1970 - 2009 (inclusive), and X^2 and p values testing for parity of the observed male:female mortality ratio.

Species	Male	Female	X^2	p
Blue whale	2	1	0.33	0.564
Bryde's whale	3	8	2.27	0.132
Fin whale	73	66	0.353	0.553
Humpback whale	108	107	0.00465	0.946
Minke Whale	72	106	6.49	0.011
Right whale	39	46	5.12	0.024
Sei whale	10	3	3.77	0.052
Sperm whale	75	58	2.17	0.14
