# Supplementary material for: Assessment of Management to Mitigate Anthropogenic Effects on Large Whales
Source: Conserv Biol. 2012 Oct 1;27(1):121–33. doi: 10.1111/j.1523-1739.2012.01934.x (PMC3562480; doi:10.1111/j.1523-1739.2012.01934.x)
Supplement: Supplementary file 3 [file cobi0027-0121-SD3.pdf]

Table S3. Determined cause of death in large whales observed 1970 - 2009 (inclusive), separated by sex and age class, where known. COD = Cause Of Death, EN = Entanglement, VS = Vessel Strike, ENVS = Entanglement and Vessel Strike, OH = Other human interaction, NH = Non-human interaction; see text for definition of categories.

COD	Total	Male	Female	Undetermined	Calf	Subadult	Adult	Undetermined
EN	323	59	68	196	51	115	35	122
VS	171	56	77	38	29	97	21	24
ENVS	5	1	3	1	2	3	0	0
OH	3	1	1	1	1	1	1	0
NH	248	116	91	41	98	103	29	18
