# Supplementary figures and images for: Assessment of Management to Mitigate Anthropogenic Effects on Large Whales
Source: Conserv Biol. 2012 Oct 1;27(1):121–33. doi: 10.1111/j.1523-1739.2012.01934.x (PMC3562480; doi:10.1111/j.1523-1739.2012.01934.x)

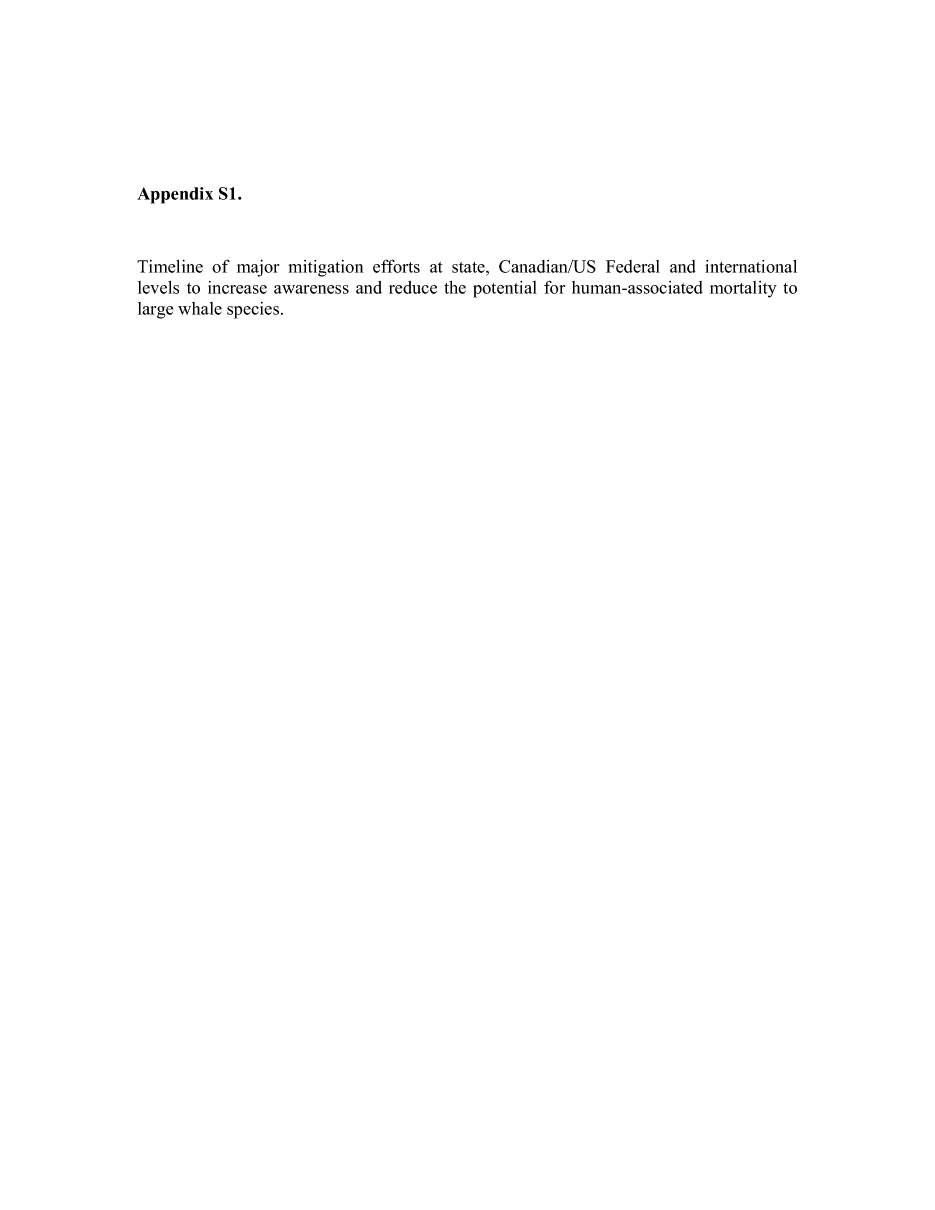

Supplement: Supplementary file 4 [file cobi0027-0121-SD4.png]

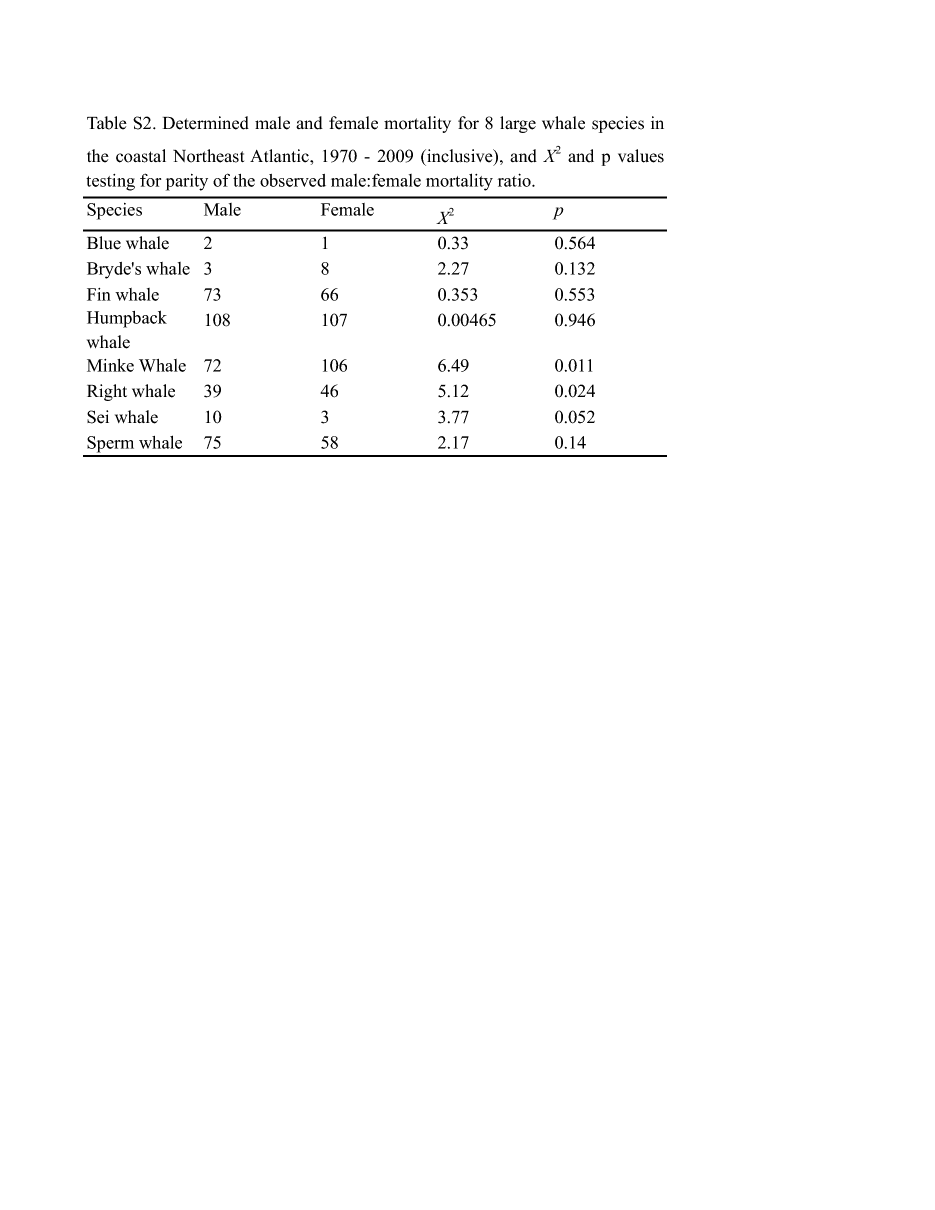

Supplement: Supplementary file 5 [file cobi0027-0121-SD5.png]

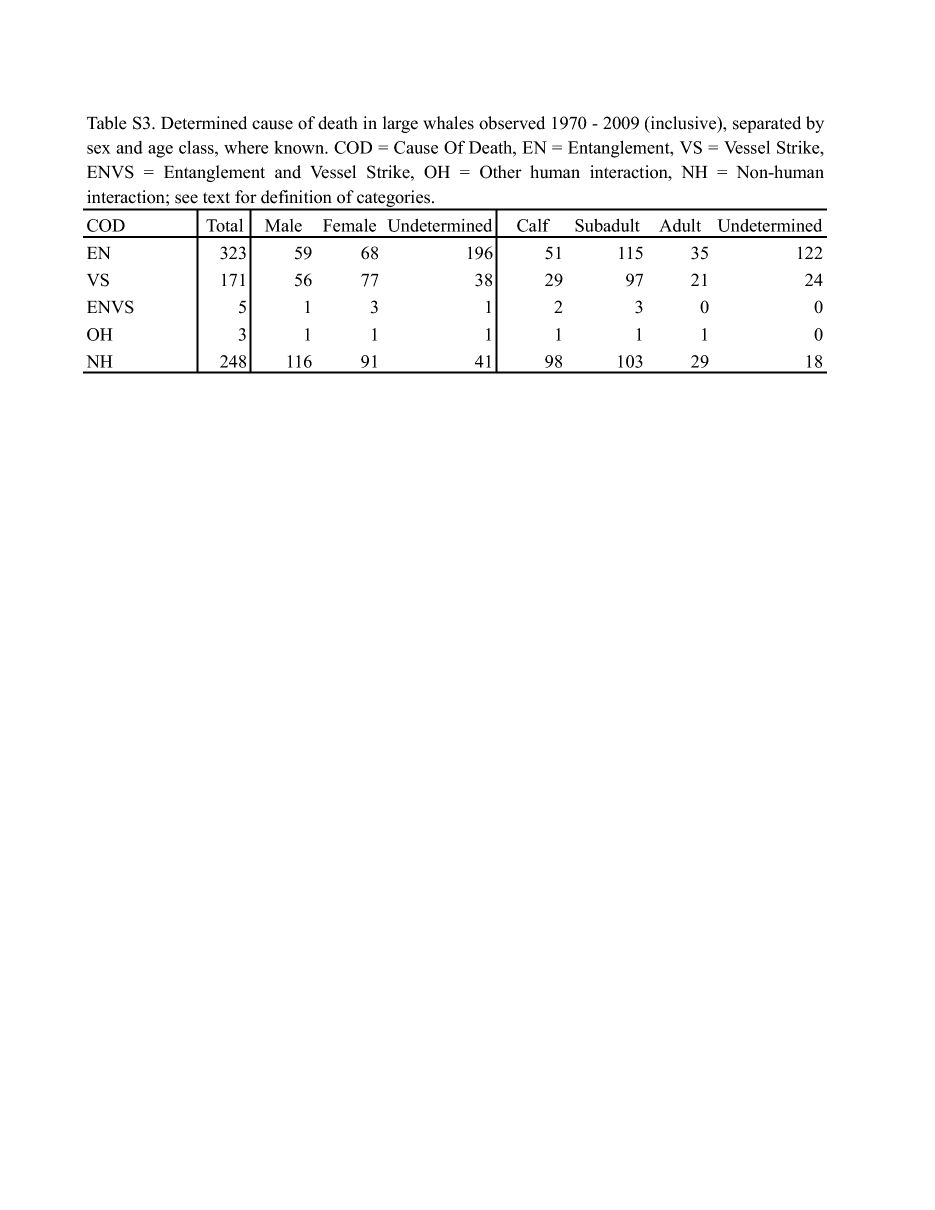

Supplement: Supplementary file 6 [file cobi0027-0121-SD6.png]
